# International football players with cerebral palsy maintained their physical fitness after a self-training program during the COVID-19 lockdown

**DOI:** 10.7717/peerj.13059

**Published:** 2022-03-17

**Authors:** Iván Peña-González, José Manuel Sarabia, Agustín Manresa-Rocamora, Manuel Moya-Ramón

**Affiliations:** 1 Department of Sport Sciences, Universidad Miguel Hernández de Elche, Elche, Alicante, Spain; 2 Alicante Institute for Health and Biomedical Research (ISABIAL Foundation), Alicante, Spain

**Keywords:** CP football, Physical performance, Training, Sport, Disability

## Abstract

**Background:**

The COVID-19 global pandemic caused a complete stop in sport participation which meant a detraining period for athletes. High-level athletes had to train at home guided by their coaches and conditioning trainers in an effort to maintain their physical fitness. The aim of maintaining the training adaptations and physical fitness during the COVID-19 mandatory lockdown was especially important for CP athletes, in which the detraining period was expected to cause early declines in motor function, poor coordination and muscle weakness due to their disability.

**Methods:**

The present study assessed the effect of a guided self-training program on international CP football players’ physical fitness during the COVID-19 mandatory lockdown. Fifteen CP football players from the Spanish National Team participated in the study. An experimental design with a pre- (T1) and a post-intervention (T2) assessment was carried out, with a 12-week period of players’ self-training (divided in two periods of 6 weeks) which combined strength and endurance training. Physical performance assessment consisted in the free countermovement jump (CMJ), 5, 10 and 20-m sprint, the modified agility T-test (MAT) and a dribbling test. The Kruskal–Wallis test was used for between-group comparisons, while the Student’s paired *t* test or the Fisher Pitman permutation test, based on the normality of the data, were used for within-group comparisons.

**Results:**

The results showed no differences between sport classes (FT1, FT2 and FT3) in physical fitness change after the training program (*Chi^2^* = 0.16 to 1.73; *p* = 0.42 to 0.92). Within-group comparisons showed an increase of jump height in the CMJ (4.19 cm [2.46, 5.93]; *p* < 0.001) and a maintenance of the 5, 10 and 20-m sprint, MAT and dribbling ability (<0.01 to 0.09 s; *p* = 0.19 to 0.97).

**Discussion:**

To the authors’ knowledge, this is the first study that examined the physical fitness adaptations to a training program with CP football players. The results show that a 12-week guided self-training program without football-specific stimulus may be effective to maintain or even improve the specific physical performance of international CP football players during a non-competitive period (as the COVID-19 lockdown). This study reveals that CP football players are able to show adaptations to the strength and endurance training and this could be the basis for future research regarding training adaptations in CP football players.

## Introduction

Football played by athletes with cerebral palsy (CP) or acquired brain injury, also called CP football, is a world-wide practiced modality of seven-a-side football. They play two 30-min halves in a 70 × 50-m field, following the International Federation of Cerebral Palsy Football (IFCPF) rules. Players must have a minimum impairment for hypertonia, athetosis or ataxia (HAA) according to the IFCPF Classification Rules (IFCPF, 2018) to be eligible for CP football. Eligible players are grouped into three sport classes depending on their level of impairment and activity limitation for the performance of basic football skills (severe impairment (FT1); moderate impairment (FT2); mild impairment (FT3) based on a sport-specific classification system (IFCPF, 2018)). The differences in activity limitations according to the CP profile, in CP football players, are mainly in coordination, balance, range of motion and symmetry (Roldan et al., 2020). Specific examples of the qualitative description of activity limitation in these categories, according to the CP profile, are provided in Roldan et al. (2020). The classification process is crucial in CP football as it considers the players impairment limitations on the sport performance and it has an impact on how to apply game rules, since during the match, teams are allowed to have only one FT3 player and they must have at least one FT1 player on the field.

CP football is an intermittent sport characterized by short and high-intensity actions (*i.e*., jumps, sprints or rapid changes of direction), which requires a high development of the anaerobic system, alternated with low-intensity or recovery periods (Yanci et al., 2016). These anaerobic and high-intensity actions are related to strength and power development of the lower limbs and they have a special importance in CP football as they are decisive in the sport performance (Yanci et al., 2016). In the last years, cross-sectional researches have studied the physical fitness characteristics of CP football players (Daniel et al., 2020; Peña-González et al., 2020; Reina et al., 2016, 2017, 2018; Yanci et al., 2021) with a special focus on how the level of impairment is related to the players’ performance with the aim of improving the evidence-based classification system. The most used physical tests for the assessment of CP football players’ physical fitness are linear sprint, vertical jump, the change of direction ability (CODA) or the dribbling ability (Pastor et al., 2019; Reina et al., 2016, 2017, 2018; Yanci et al., 2014), which have been commonly used to differentiate between CP football players with different activity limitation levels. Nowadays, there is an increasing interest to research how these physical fitness variables may have an impact on CP football talent identification, selection and long-term development processes (Peña-González et al., 2021).

Furthermore, the coronavirus disease 2019 (COVID-19) global pandemic caused a complete or partial halt in the sport calendar during 2020. One of the strategies used by countries with a high incidence of the disease (*e.g*., Spain) was to apply a mandatory lockdown that forced people to remain at home and consequently elite athletes were forced to modify their training routines (Sarto et al., 2020). Although the literature that examines how the training process produces physical adaptations in CP footballers is scarce, the training process in CP athletes seems to be crucial to maintain their physical fitness values as the CP disability causes an early decline in motor function, and poor coordination and muscle weakness are the main limitations of CP population (Cans, 2000), as well as of CP football population (Reina et al., 2016) who have lower levels of impairment compared to general population with CP (Palisano et al., 2008). For CP football players, who follow systematic training processes, the effects of a detraining period (*e.g*., a lockdown) are not known, but the same as in regular football, negative consequences in their physical fitness can be expected (Christensen et al., 2011; Joo, 2018). Due to these expectations of a possible physical fitness detriment during the lockdown period, the main aim of the athletes worldwide during this period was to maintain their general fitness and health (Washif et al., 2021). However, less than the 40% of the athletes were able to maintain their sport-specific training (including national, international and world-class athletes) (Washif et al., 2021). With the aim of reducing the effects of detraining in physical fitness and trying to maintain the level of physical activity, coaches and conditioning trainers designed self-training protocols to implement with their CP football players during the lockdown period. The maintenance of specific physical adaptation to CP football requirements by performing an individual (at home) self-training, without any sport-specific training stimulus, was a challenge for the CP football collective.

In this regard, previous literature has addressed the topic of how different training programs may have an impact on physical fitness in CP population (Gillett et al., 2018; Scholtes et al., 2010; Taylor et al., 2013). Most of these approaches were based on strength training because the impairment is related to muscle weakness due to alterations in structural and neurological muscle features (Fleeton, Sanders & Fornusek, 2020). Various studies included in the systematic review conducted by Fleeton, Sanders & Fornusek (2020) have reported physical adaptations to different strength-training programs but these studies were carried out with a non-athlete CP population. In addition, most of these included studies were carried out with a non-ambulatory and highly affected CP population (levels 2–5 of the Gross Motor Function Classification Scale (GMFCS)) (Palisano et al., 2008) while CP football players are characterized by a low activity limitation (level one of GMFCS). In this regard, a major concern for coaches and conditioning trainers in CP football are the possible differences in training adaptations between players with different levels of impairment, which may mean different training methodologies among team players.

To the authors’ knowledge, there is no research that specifically examines the physical fitness adaptations to a training program in CP football (Fleeton, Sanders & Fornusek, 2020). Therefore, considering the lack of knowledge about the physical adaptations to a training program in CP football players and the possible negative effects in players’ physical fitness due to the halt in their training processes produced by the COVID-19 mandatory lockdown in Spain, the aim of this study was to assess the effect of a self-training program on the physical fitness of international CP football players during the lockdown period.

## Materials and Methods

### Participants

Fifteen CP football players from the Spanish National Team participated in the study. Descriptive characteristics of the overall sample and based on the sport class can be found in Table 1. No participant suffered any type of injury during the whole study and all of them attended the two testing sessions. All the players were registered in the Spanish Sports Federation of People with Cerebral Palsy (FEDPC). Since the participants were from different Spanish cities, and considering that this study was carried out in an ecological environment, the participants performed a physical self-training program following the researchers’ guidelines. Each participant signed an informed consent according to the Declaration of Helsinki (2013). The protocol of this study was approved by the ethical committee of the Miguel Hernández University (Reference number CID.DPC.01.19).

**Table 1:** Descriptive characteristics in overall players and based on the class.

	Overall (*n* = 15)	FT 1 (*n* = 4)	FT 2 (*n* = 9)	FT 3 (*n* = 2)	
Age (years)	21.0 [18.0–30.0)	29.5 [20.8–38.3]	21.0 [18.0–25.5]	18.0 [16.0–20.0]	
Height (cm)	174.0 [168.0–182.0]	169.5 [164.3–180.8]	177.0 [168.0–182.0]	175.0 [169.0–181.0]	
Weight (kg)	64.0 [54.6–78.9]	69.9 [53.7–90.9]	63.6 [56.4–73.6]	67.5 [66.1–68.8]	
BMI (kg·m^−2^)	20.6 [19.9–23.6]	23.9 [19.9–28.2]	20.6 [19.5–23.1]	22.1 [21.0–23.1]	
Experience (years)	4.5 [3.5–6.5]	4.5 [2.8–12.3]	4.5 [2.5–7.0]	6.0 [5.5–6.5]	

**Note:**

BMI, body mass index; *n*, number of included players; Values are presented as median (25^th^ and 75^th^ percentiles).

### Design and procedure

An experimental research design with a pre-post intervention was carried out to examine the effect of a self-training program carried out with CP football players during the COVID-19 mandatory lockdown period. The subjects were instructed not to perform any other type of physical activity during this period and the researchers supervised the training sessions by means of group videocalls to ensure the correct development and exercise execution in each session.

A guided (non-football specific) training program was provided to the participants to be performed individually (self-training). To analize the effect of this program during the specific COVID-19 lockdown period, the last testing session before to the COVID-19 lockdown was used as testing session 1 (T1) in this study. The testing session 2 (T2) was located as soon as the Spanish government allowed group gatherings, 12 weeks after the T1. Each testing session was separated from the previous and following training session by at least 48 h. Since the present study was carried out in an ecological environment, the authors did not consider it ethical to include a control group which did not perform the physical fitness training program. Since the time of the lockdown was not previously established, this training program was updated on a weekly basis. The final duration of the self-training program was 12 weeks and it finished when the Spanish government allowed group gatherings and it was possible to carry out the T2. The first 7 weeks (period 1), the participants were not allowed to leave their home, thus only sided exercises were performed. During the following 5 weeks the Spanish government allowed elite players to carry out physical activity outdoors individually. For this second period (period 2), running-based trainings were included. The training program consisted in four training sessions per week, and it is detailed in Table 2.

**Table 2:** Descriptive self-training program.

Period 1 (weeks 1 to 6)						
Monday	Tuesday	Wednesday	Thursday	Friday	Saturday	Sunday
*6 exercises* *4 bouts X exercise* *12 rep X bout* *30 s. rest X bout* *1 min. rest X exercise*		*6 exercises in circuit* *4 rounds to circuit* *30 s. work* *30 s. rest* *1 min. rest X round*	*6 exercises* *4 bouts X exercise* *12 rep X bout* *30 s. bout rest* *1 min. exercises rest*		*6 exercises in circuit* *4 rounds to circuit* *30 s. work* *30 s. rest* *1 min. rest X round*	
Squat		Low skipping	Squat		Low skipping	
Lunge		Lateral sided jumps	Lunge		Lateral sided jumps	
Side lateral lunge		Repeated CMJs	Side lateral lunge		Repeated CMJs	
Hip thrust		High skipping	Hip thrust		High skipping	
Dead lift		Triple hop	Dead lift		Triple hop	
Ankle plantar flexion		Burpees	Ankle plantar flexion		Burpees	
**Period 2 (week 7 to 12)**						
**Monday**	**Tuesday**	**Wednesday**	**Thursday**	**Friday**	**Saturday**	**Sunday**
*6 exercises* *4 bouts X exercise* *12 rep X bout* *30 s. rest X bout* *1 min. rest X exercise*		*4 bouts X 4 min. high intensity running* (*>80% FCmax*) *3 min. rest X bout*	*6 exercises in circuit* *4 rounds to circuit* *30 s. work* *30 s. rest* *1 min. rest X round*		*2 blocks X 6 bouts X 30 s. all out running* *30 s. rest X bout* *2 min. rest X block*	
Squat			Low skipping			
Lunge			Lateral sided jumps			
Side lateral lunge			Repeated CMJs			
Hip thrust			High skipping			
Dead lift			Triple hop			
Ankle plantar flexion			Burpees			

**Note:**

CMJs, Countermovement jumps.

### Measurements

The players were asked about their age and experience directly. The players’ body height and weight were assessed using a fixed stadiometer (SECA Ltd., Germany ± 0.1 cm) and a scale (Tanita Bc 601 Ltd., India ± 0.1 kg), respectively. Their body mass index (BMI) was calculated as weight (kg) · (height (m))^−2^. The physical fitness test battery consisted in: (1) a free arm swing countermovement jump (CMJ); (2) the time taken to run a 5, 10 and 20-m linear sprint; (3) a modified agility T-test (MAT) (Sassi et al., 2009) performing only forward displacements (which are more common in football) and removing the rule about “touching the cone” because of the participation of athletes with upper limbs affected by spasticity (Arcos et al., 2020); and (4) a dribbling test with the same structure of the MAT (Peña-González et al., 2021). Players were already familiarized with physical fitness tests because they periodically took part in the long-term monitoring group of FEDPC. Before the assessment of the physical fitness tests, players carried out the same standardized warm-up described for the training sessions including 3-min of high intensity actions as jumps, sprints or changes of direction. The jump height in the CMJ was estimated using a contact platform (Globus Ergotester®, Italia). The time taken to perform the linear sprint, the MAT and the dribbling test was recorded using a photocell system (Witty System, Microgate, Bolzano, Italy). Players performed two attempts of each physical fitness test with a 2-min rest between each attempt and the best attempt was recorded for the further analysis. Sprint, MAT and dribbling tests were carried out on a synthetic-grass football pitch and players wore their usual football boots. Players were verbally encouraged to perform at their maximal effort during the physical fitness tests.

### Statistical analysis

Within-session relative (ICC) and absolute (SEM) reliability analysis were carried out for the CMJ, 5, 10 and 20-m linear sprint, MAT and dribbling tests. For the interpretation of ICC values, values >0.90 were considered as excellent, values from 0.75 to 0.90 as good and values <0.75 were considered as poor to moderate (Portney & Watkins, 2002). The SEM was shown in percentages and it was calculated as: SEM% = (SEM/mean) × 100. The Shapiro–Wilk test, box plot and Q-Q graphs were used to test the normality of the data. Based on the normality assumption, data at pre- and post-intervention, as well as changes at post-intervention were reported as mean ± standard deviation or median and 25th and 75th percentiles. Percentiles were calculated by the weighted average method. The Kruskal–Wallis test with chi-squared statistic was used to carry out between-group (*i.e*., sport class) comparisons in baseline characteristics and changes at post-intervention. Based on the normality assumption of the change at post-intervention in each variable, the Student’s paired t test or the Fisher Pitman permutation test were used to perform within group comparisons. Parametric or non-parametric approaches were also used to estimate the 95% confidence interval around the mean or median change (Hodges & Lehmann, 1963) respectively. The standardized differences or effect sizes (ES) at 95% CI between sport classes were expressed in Cohens’ d units and they were interpreted as trivial (<0.19), small (0.20–0.49), moderate (0.50–0.79) and large (>0.80) (Cohen, 1992). Statistical significance was set at *p* ≤ 0.05. All analyses were performed using STATA software (version 16.0; Stata Corp LLC, College Station, TX, USA).

## Results

**Figure 1:**
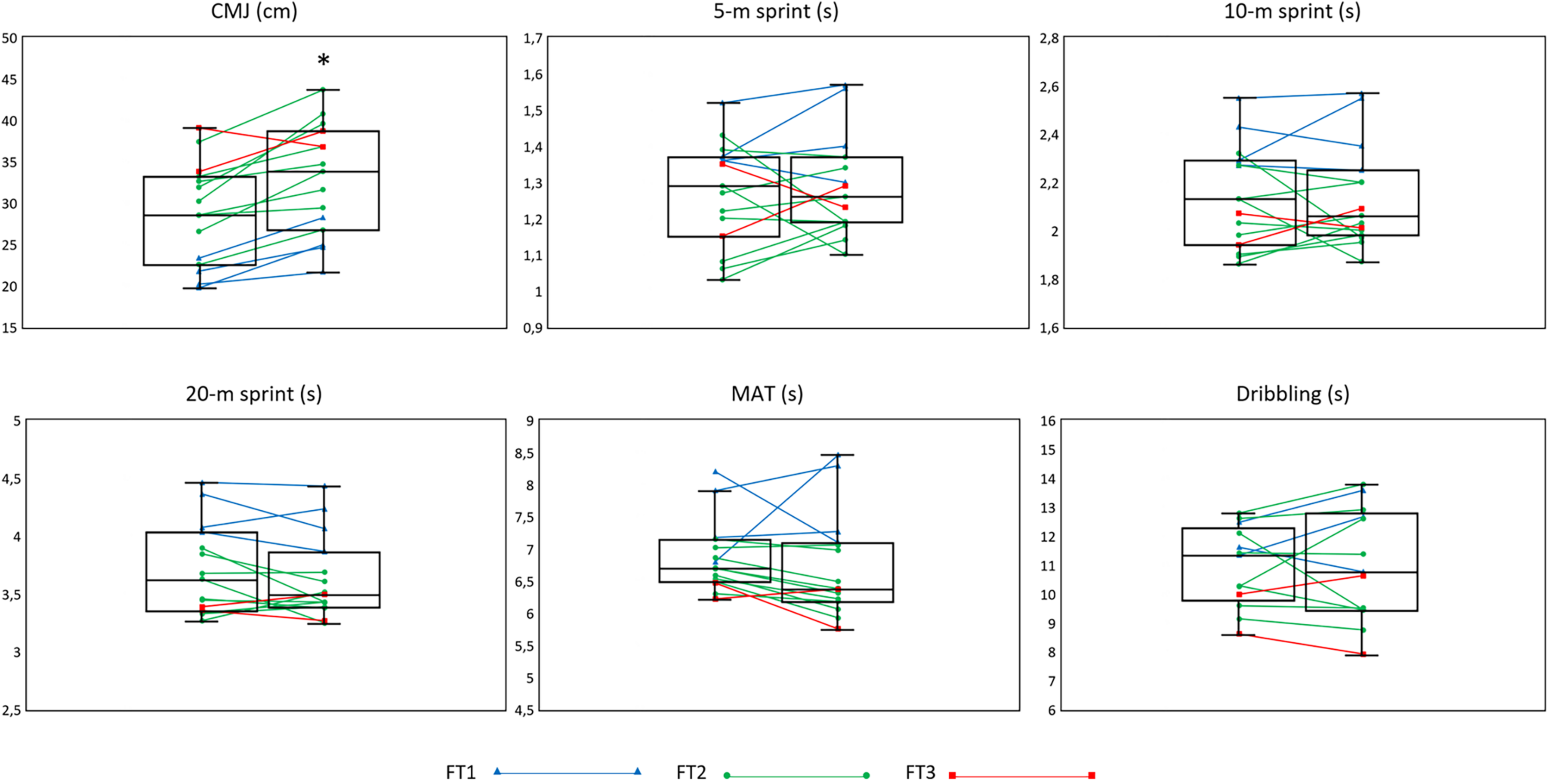
Average and individual changes in physical fitness between T1 and T2. *p < 0.05 (significant difference between T1 and T2 for the average of the data).

Physical performance tests used in this study presented excellent reliability values (CMJ: ICC = 0.92, SEM = 6.78%; 5-m sprint: ICC = 0.94, SEM = 3.43%; 10-m sprint: ICC = 0.95, SEM = 2.72%; 20-m sprint: ICC = 0.98, SEM = 1.93% and MAT: ICC = 0.95, SEM = 2.00%) except for the dribbling test which presented good values (ICC = 0.79, SEM = 6.90%). The between-group comparison showed no statistically significant differences among sport classes in the median changes reached at post-intervention in any of the analysed physical fitness tests (*Chi*^*2*^ = 0.16 to 1.73; *p* = 0.42 to 0.92) (Table 3). Therefore, the training-induced effects on the physical fitness tests were examined regardless of the sport class. The Student’s paired t test or the Fisher Pitman permutation test (depending on the normality of the data), as well as the ES, showed a maintenance in players’ physical fitness between pre- and post-testing sessions (mean changes between <0.01 and 0.09 s in the 5, 10 and 20-m sprint, the MAT and the Dribbling tests; *p* = 0.19 to 0.97) (Table 4). In addition to this, the CMJ had a statistically significant enhancement between pre- and post-testing sesions (mean change: 4.19 [2.46, 5.93] cm ; *p* < 0.01). The individual percentage of change is shown in Fig. 1 for each player in each physical fitness test, including the mean percentage of change.

**Table 3:** Between-group comparisons for the change reached at post-intervention.

Test	Sport class	*n*	Change	*Chi* ^*2*^	*p*
CMJ (cm)	FT1	4	3.90 [1.85–5.13]	1.36	0.507
	FT2	9	4.20 [2.60–7.50]		
	FT3	2	1.30 [−2.30 to 4.90]		
5-meters (s)	FT1	4	0.045 [−0.035 to 0.155]	0.22	0.895
	FT2	9	0.040 [−0.105 to 0.095]		
	FT3	2	0.010 [−0.120 to 0.140]		
10-meters (s)	FT1	4	0.000 [−0.065 to 0.200]	0.16	0.922
	FT2	9	0.050 [−0.165 to 0.085]		
	FT3	2	0.045 [−0.060 to 0.150]		
20-meters (s)	FT1	4	−0.100 [−0.268 to 0.113]	0.28	0.869
	FT2	9	−0.020 [−0.310 to 0.075]		
	FT3	2	0.010 [−0.090 to 0.110]		
MAT (s)	FT1	4	0.240 [−0.810 to 1.350]	1.73	0.422
	FT2	9	−0.310 [−0.460 to −0.145]		
	FT3	2	−0.285 [−0.720 to 0.150]		
Dribbling (s)	FT1	3	1.110 [−0.850 to 1.340]	0.46	0.795
	FT2	8	−0.060 [−0.725 to 0.825]		
	FT3	2	−0.030 [−0.700 to 0.640]		

**Notes:**

*Chi*^*2*^, Chi-squared statistic; CMJ, Countermovement jump; *n*, number of players included in the analysis; *p*, probability level associated to the *chi*^*2*^ statistic.

Changes are presented as median (25^th^ and 75^th^ percentiles).

**Table 4:** Training-induced effect on the analysed variables in the overall sample.

Test	Pre	Post	*p*	Change (95% CI)
CMJ (cm)	28.60 ± 6.18	32.79 ± 6.69	<0.001	4.19 [2.46–5.93]
5-m sprint (s)	1.272 ± 0.145	1.287 ± 0.141	0.640	0.015 [−0.053 to 0.084]
10-m sprint (s)	2.137 ± 0.211	2.139 ± 0.213	0.974	0.001 [−0.089 to 0.089]
20-m sprint (s)	3.698 ± 0.392	3.625 ± 0.361	0.188	−0.073 [−0.187 to 0.040]
MAT (s)	6.69 (6.48; 7.134)	6.37 (6.17; 7.09)	0.384	−0.022 [−0.488 to 0.048]
Dribbling (s)	10.91 ± 1.39	11.01 ± 1.95	0.798	0.092 [−0.671 to 0.854]

**Note:**

CI, confidence interval; CMJ, Countermovement jump; *n*, number of players included in the analysis; *p*, probability level associated to the Student’s paired t test or Fisher-Pitman permutation test. Values at pre- and post-intervention are delivered as mean ± standard deviation or median (25^th^ and 75^th^ percentiles).

## Discussion

The aim of this study was to assess the effect of a 12-week self-training program on the physical fitness in international CP football players during the COVID-19 mandatory lockdown period. To the authors’ knowledge, this is the first study that reports the effects of a self-training program in CP football players. The main finding of this study suggests that a 12-week general self-training program, with four training sessions per week, may contribute to maintain (or even improve) football-specific physical fitness during a non-competitive period. This main finding is important since it has been recently reported that less than the 40% of athletes worldwide were able to maintain their sport-specific training and the main aim of the athletes was in an 80% to “maintain their general fitness and health” (Washif et al., 2021).

In the practical field, there is a lack of consensus whether the conditioning process should be different for CP football players according to their sport class. In this regard, previous literature has shown physical fitness differences between the three sport classes of CP football (*i.e*., FT1, FT2 and FT3) in favor of the higher sport classes (Peña-González et al., 2020). Although the results showed in Table 4 and Fig. 1 could suggest that CP football players from different sport classes may have similar adaptations to a training program, these results should be taken with caution due to the small and unbalanced sample size included in this study.

This 12-week general self-training program with four training sessions per week improved the CP football players’ jump ability (4.19 cm of mean change; *p* < 0.001), while their acceleration, sprint velocity, change of direction and dribbling performances remained the same. Figure 1 shows the high tendency of the CMJ to improve after the training program in all the players but trivial increases/decreases were seen in the rest of the physical fitness tests. Several studies have shown jumping improvements with similar training methodologies in a wide spectrum of ages in able-bodied football players (Asadi et al., 2018; Manouras et al., 2016; Peña-González et al., 2019). Although jumping performance is lower in football players with cerebral palsy compared to able bodied football players (Yanci et al., 2014), the results of this study seem to indicate that CP football players are able to show training adaptations that improve their physical fitness. This idea is in line with that by Gillett et al. (2016), who reported that muscles of people with CP are able to adapt to the training, mainly due to morphological and architectural adaptations. However, it is well documented in people without CP that neuromuscular and coordinative adaptations (*i.e*., improvements in the stretch-shortening cycle (SSC) function) are the main responsible for improvements in football-specific actions such as jumping. Nevertheless, there is no information about the effect of training on the SSC function in CP population. The literature has shown benefits in the muscular function (*i.e*., reducing quadriceps/hamstring co-contraction or improvements in an agility test) when people with CP who were not sport samples but exercised with rehabilitation purposes (spastic hemiplegia) performed a plyometric-based strength training (Elnaggar, 2020; Johnson et al., 2014).

The CP football players’ physical fitness in the 5, 10 and 20-m sprint, change of direction and dribbling was maintained after the self-training program of this study. A physical fitness decrement after a detraining period has been widely studied in football (Christensen et al., 2011; Joo, 2018; Koundourakis et al., 2014), but in this study, the “detraining” period was a mandatory lockdown in which the technical staff tried to maintain the players’ physical fitness through a self-training at home. A previous study by Nakamura et al. (2012) showed no differences in football players’ physical fitness after a detraining period between players who did not train, players who performed an endurance training program twice a week and players who performed a plyometric program twice a week (Nakamura et al., 2012). In contrast, Christensen et al. (2011) showed physical fitness maintenance after a 2-week period in which football players performed a high-intensity training program while players who stopped their activity reduced their values in the same variables. However, we cannot compare our results with the mentioned study because in their training program, players performed football-specific tasks (*i.e.*, small-sided games), which the participants of the present study were not allowed to carry out due to the mandatory lockdown. This is directly related to the training specificity principle, as reported by Rodríguez-Fernández et al. (2020) who suggest that only specific training, based on small-sided games and repeated sprint ability allow football players to maintain specific adaptations to football performance (Rodríguez-Fernández et al., 2020). Nevertheless, the results of the present study suggest that the general training program carried out with CP players during the lockdown period was effective to maintain their physical fitness during a non-competitive period. In addition, statistical improvements in the CMJ but not in the other physical fitness tests may be explained by the prescription of strength exercises mainly with vertical force production, which may mean a limitation for the present training program. It has been observed in previous literature that it is necessary to incorporate exercises with greater horizontal force production in the strength trainings to enhance acceleration and sprint performance (Sáez de Villarreal, Requena & Cronin, 2012).

This study has some limitation that should be considered. The training program consisted mainly in strength exercises with vertical instead of horizontal force production, which may have contributed to the higher CMJ improvements than in the other physical tests. Future research in the field of physical training with CP football players should include a control group to compare the physical fitness adaptations to the training, and the training protocol may include some exercises with horizontal force application as well as include endurance training and evaluation.

## Conclusions

This study shows a 12-week training program performed individually (self-training) by international CP football players, which contributed to maintain, or even improve, football-specific physical fitness during a non-competitive period. The combination of general (not football-specific) strength and endurance exercises included in the four sessions per week helped CP football players to maintain football-specific physical fitness which may be important in non-competitive periods.

## Supplemental Information

10.7717/peerj.13059/supp-1 Supplemental Information 1 Dataset.

## Additional Information and Declarations

### Data Availability

The following information was supplied regarding data availability: The raw measurements are available in the Supplemental File.
